# Fiber Optic Sensing Textile for Strain Monitoring in Composite Substrates

**DOI:** 10.3390/s22239262

**Published:** 2022-11-28

**Authors:** Andres Biondi, Rui Wu, Lidan Cao, Balaji Gopalan, Jackson Ivey, Camila Garces, Michael Mitchell, John D. Williams, Xingwei Wang

**Affiliations:** 1 Department of Electrical and Computer Engineering, University of Massachusetts Lowell, Lowell, MA 01854, USA; 2 Saint-Gobain Research North America, Northborough, MA 01532, USA; 3 The Boeing Company, Huntsville, AL 35808, USA

**Keywords:** structural health monitoring, distributed fiber optics, smart textile, composites, optical frequency domain reflectometry (OFDR), smart structures

## Abstract

Composite polymers have become widely used in industries such as the aerospace, automobile, and civil construction industries. Continuous monitoring is essential to optimize the composite components’ performance and durability. This paper describes the concept of a distributed fiber optic smart textile (DFOST) embedded into a composite panel that can be implemented during the fabrication process of bridges, planes, or vehicles without damaging the integrity of the composite. The smart textile used an embroidery method to create DFOST for easy installation between composite laminates. It also allows different layout patterns to provide two- or three-dimensional measurements. The DFOST system can then measure strain, temperature, and displacement changes, providing critical information for structural assessment. The DFOST was interrogated by using an optical frequency domain reflectometry (OFDR). It could measure strain variation during the dynamic and static test with a spatial resolution of 2 mm and a minimum strain resolution of 10 μϵ. This paper focuses on the study of strain measurement.

## 1. Introduction

Fiber-reinforced polymer (FRP) composite has become a popular material for the automotive, aerospace, and civil industries thanks to its excellent properties, such as its light weight, robustness, durability, corrosion resistance, and the capability to mold large, complex shapes in a short cycle time [1,2,3,4,5]. Additionally, it is cost-efficient and environmentally friendly. For example, fabricating aircraft, bridges, and automobiles by using composite materials will reduce the overall weight of the final design. Lightweight planes, airplanes, and vehicles result in less fuel consumption, which in turn results in economic savings and a reduction of environmental pollution.

Nevertheless, FRP structures and components are susceptible to damage due to delamination, matrix cracking, inter-laminar fracture, and debonding. They are also susceptible to impact damage, with a strong possibility of internal damage going unnoticed. Therefore, it is necessary to inspect FRP structural elements to avoid any catastrophic failure. Due to the composite material’s microstructure nature, detecting damage by using visual inspection is not ideal. For this reason, different nondestructive monitoring methods have been developed to track changes in the composite material, such as strain, cracks, and temperature. These techniques include measuring acoustic emission during cracking, deformation, debonding, and impact [6,7,8]. Thermal imaging methods are used to detect a surface defect or anomalies by measuring the temperature differences observed in an investigated surface by using infrared sensors and cameras [9]. The ultrasonic method is used to inspect adhesive-bonded multilayered structures with laminated composite components that monitor delamination and interlaminar weakness [10]. New techniques have also been developed, and they include microelectromechanical sensors (MEMS) [11], and nanoelectromechanical systems (NEMS) [12].

These techniques are great for detecting localized damage or local abnormalities in the structures. However, point-based sensors cannot provide complete monitoring of a large structure. This is because point base sensors can only provide information from one particular location, which does not contain enough information to assess the complete structure. Multiple sensors can be installed, but this will require a longer installation time and multiple signal cables to transmit the information of any single sensor, making it too bulky for large structures. Additionally, the large size of the point-based sensors limits the ability to embed them into the composite because it can negatively affect the performance of the composite part. Therefore, new measurement techniques are being constantly studied to improve the ability to detect and provide comprehensive information. Currently, distributed fiber optic sensing (DFOS) [13,14,15] has shown great potential to be implemented in large structures because it enables the replacement of thousands of traditional strain gauges with the use of a single fiber cable. Additionally, DFOS not only has the ability to monitor strain values but also can monitor other physical properties such as temperature [16,17], pH changes [18], vibration [19], displacement, and shape [20,21]. With the different types of information DFOS can provide, this technology can be implemented across different industries. For example, in the area of civil engineering, DFOS can monitor structures with concrete [22,23,24,25] and steel [26]. For the aerospace and automobile industry, DFOS can monitor FRP components [27,28,29,30,31].

DFOS can be categorized as quasidistributed and fully distributed sensors. Quasidistributed sensors are based on Bragg Grating (FBG), which are microstructure-inscribed in the core of a single-mode fiber to generate an interference pattern. FBG has been used to monitor different types of physical properties such as temperature, humidity [32], and strain [33,34]. They provide a great advantage in monitoring events in extreme environments due to their survivability and sensitivity. However, there are several drawbacks to this type of sensor. First, FBG should be designed to guarantee a measurable wavelength range of the interrogation system and no spectra overlapping in the measurable wavelength domain [35]. Secondly, the number of FBG in a single-fiber cable is limited by the maximum strain value the fiber sensor will experience [36]. Lastly, FBG fabrication also entitles high labor costs because of the required equipment and personnel, limiting the mass production and the cost-effectiveness [37,38].

Fully distributed fiber optic sensors (DFOS) have improved some of the drawbacks of quasidistributed sensors. The main advantage is removing the limitation in the number of sensors the fiber cable can carry. With DFOS the entire fiber becomes a sensor with multiple sensing locations, allowing the measurement of large amounts of information. Additionally, there is no need to inscribe microstructure in the fiber core; regular communication cables can be used as sensing devices. There are multiple interrogation methods used in DFOS. They are all based on the scattering process inside the fiber core and can be classified as Rayleigh, Brillouin, and Raman distributed sensing. Rayleigh scattering is an elastic process in which the scattered photons have the same wavelength and frequency as the incident photons [39]. The Brillouin method is an inelastic scattering process that occurs because of light propagation through fibers with material density fluctuation. Its frequency shift depends on the acoustic velocity and the fiber’s index of refraction, which is affected by changes in temperature and strain. For this reason, Brillouin-distributed sensors have been used widely to monitor strain and temperature [40,41,42]. Raman scattering is also an inelastic scattering, and it is due to the molecular vibration whereby the incident light interacting with optical phonon is scattered [43]. The intensity of the stokes is only dependent on temperature. Hence, Raman scattering is only used for temperature monitoring.

DFOS has changed completely the sensing approach used to monitor structures. Nevertheless, the main disadvantage of the DFOS system is the high cost of the interrogation systems as well as the labor cost for the fiber installation. For this reason, this paper aims to explore a new process to reduce the time and complexity of the fiber installation process. Previous works have discussed integrating DFOS inside composite parts for SHM [44]. The author used OFDR approach to interrogate fiber sensors embedded in the composite sample. However, difficulties during the fiber installation were encountered. The fiber layout process took a couple of hours, and special care was required due to the fiber breakage that could occur during the routing of the fiber. Other approaches have been done to facilitate the fiber installation. For example, fibers were integrated into a tape [45,46] to improve the installation process and protect the fiber. In these papers, the tape was installed on a bridge girder to monitor the strain change during the loading condition. This was a significant improvement for DFOS installation; however, it only allowed the fiber to be installed in one way, which means this approach was not suitable to monitor areas with complex locations and shapes. Additionally, embedding the tape into composite samples will not be possible because it will affect the performance of the composite due to its bulky size. In our previous work, an approach was developed to use an embroidering method to embed the fiber into textiles to measure temperature and strain [47]. This approach helped to improve not only the installation process but also allows the design of patterns that can be embedded in a textile as demonstrated by [41]. In this paper, distributed fiber optic sensing textile (DFOST) was designed to detect different strain directions by using one single line of fiber cable. The smart textile was attached to a pipeline, and depending on the location of the fiber, different strain information was acquired.

DFOST has the potential to be used in different areas where structural health monitoring is required, such as aerospace, civil engineering, and the auto industry. Thanks to the ability to draw fiber patterns, it can be tailored to measure strain and/or temperature in locations where it is difficult to install conventional sensing systems. For example, the textile can be embedded into airplanes’ wing to monitor its structural status after a certain amount of fly time. In the area of civil infrastructure, it can be easily attached to girders and/or be embedded during the manufacturing of composite girders. It can also be used to monitor larger structural surfaces such as dams, oil pipelines, etc.

By using the approach demonstrated in our previous work [41], this paper explored the integration of DFOST into composite parts during the autoclave process. With the use of smart textiles, we aimed to demonstrate the ability to use this technique not only for installation on the surface of a structure but also its ability to be embedded into materials such as composites. Additionally, the advantage of using DFOST in comparison with other techniques, such as fiber tape and manual installation, was demonstrated by its fast implementation and the ability to design fiber patterns. To validate the performance of the proposed textile sensor, the fabric was embedded into composite material and subjected to different laboratory experiments (e.g., cantilever test). OFDR was used as the interrogation method to measure strain responses. For further validation, the strain measurements collected by the smart textile were compared with strain gauges and fiber optic sensors installed on the surface of the composite panel.

## 2. Distributed Fiber Optics Sensing System

### 2.1. Optical Frequency Domain Reflectometry (OFDR) Interrogation System

The interrogation system used to collect the strain information from the smart textile was based on Rayleigh’s backscatter. OFDR provides a high spatial resolution of a few millimeters for tens of meters of measurable fiber length [48,49,50]. The Rayleigh backscatter is caused by random fluctuation in the index profile along the fiber. The applied strain on the fiber sensor will cause changes in the reflected spectrum. Monitoring these changes allows the fabrication of a distributed strain sensor.

The fiber’s random spectral but static scatter process can be considered as the sensor fingerprint. Similar to a standard Bragg grating, the Rayleigh backscatter responds to changes in the refractive index or physical length created by variation in strain. The relation between the spectral shift Δν and the strain variation Δϵ and the temperature ΔT is given as

(1) $$\Delta \nu =K_{T}\Delta T+k_{\epsilon }\Delta \epsilon ,$$

where $K_{T}$ and $K_{\epsilon }$ are the temperature- and strain-sensitive coefficients. A more in-depth discussion regarding the functionality of OFDR is discussed in reference [51].

### 2.2. Textile and Composite Fabrication

Before embedding the fiber into the textile, different fiber sizes were investigated to explore how the size affects the sensor sensitivity. Fiber optic cables come in different sizes and with different layers of protection. Therefore, the overall size of the fiber should not affect the properties of the composite panel. Furthermore, the fiber should withstand at least 150 °C during the autoclave process and high strain values during the testing phases. Therefore, two single-mode fibers were chosen. Both fibers with the same core size (9 μm). However, the coating material and coating diameter were different. One fiber had an acrylate and jacketed coating with a 650-μm diameter, and the second fiber had a polyimide coating with a 145-μm diameter. Polyimide coating allows the fiber to survive up to 350 °C whereas the acrylate and jacketed last between 130 °C and 200 °C depending on temperature–time exposure. This study was done for two reasons: (1) to reduce the overall cost of fiber fabrication (jacketed fibers are less expensive than polyimide) and (2) to determine how economic fibers will affect the sensitivity of strain measurements.

Both fibers were embedded into a cost-effective reinforcing fabric provided by ADFORS-Saint Gobain (XP414 laid scrim), which was made by chemically bonding continuous filament yarn in open mesh construction. The open mesh was laminated between two nonwoven substrates, and the resulting composite fabric can be used to increase tear or puncture resistance, improve dimensional stability, or aid in processing. The fiber was stitch-bonded onto the fabric by using an embroidery machine. This equipment can perform a “roll to roll” function, which allows for the production of long-length samples. A calibration test was conducted to observe the strain transfer ratio. To investigate the response, fibers stitched in the textiles were stretched by using a tensile machine, and strains were measured by using optical frequency domain reflectometry (OFDR) from LUNA Innovation. The tension on the fiber was controlled by using an AGS-X Shimadzu 10KN test frame. The sample size was 45 cm × 6 cm, and the tensile machine was set up to run for three cycles. Each cycle included the stretch and release of the textile.

**Figure 1:**
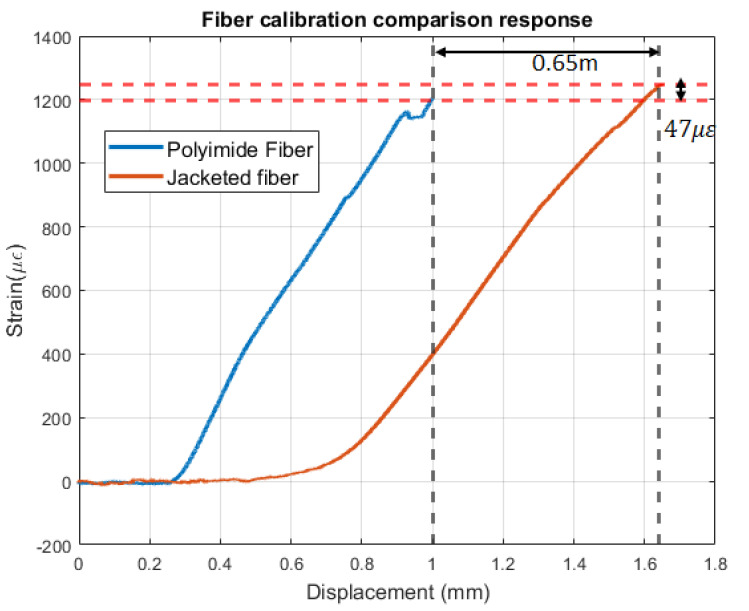
Calibration results (Strain vs. displacement).

**Figure 2:**
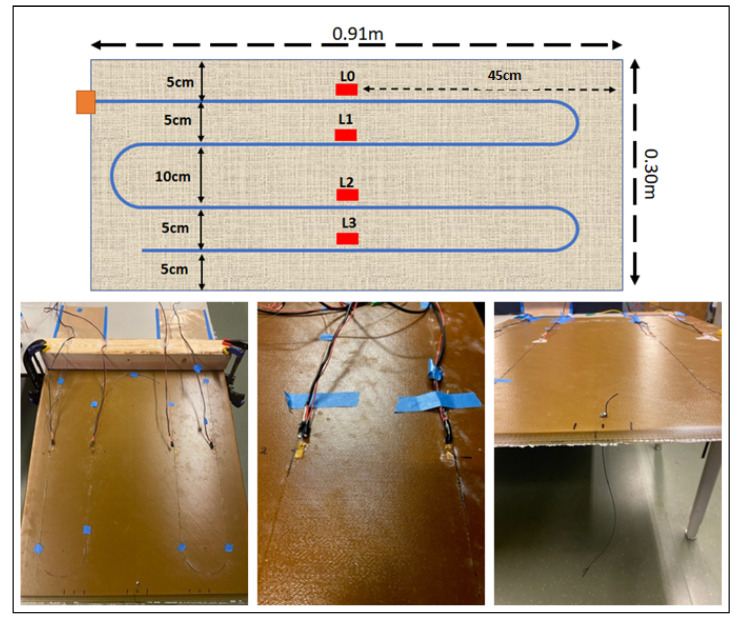
Canteliever test set up and textile layout.

A linear response was observed for both samples Figure 1. The polyimide and jacketed fiber strain coefficients were 1746 μϵ/mm and 1308 μϵ/mm, respectively. These results demonstrated that polyimide fiber has a better response to strain changes. The added jacketed coating reduces the strain transfer to the fiber’s core. Additionally, polyimide fiber started detecting strain changes at a 0.2-mm displacement while the jacketed was at 0.4 mm. This further confirms that polyimide has a better strain response; hence, it is better suited for the application explored in this research. In addition, the polyimide fiber had a size comparable to the fiber present in the composite sample. Therefore, it did not affect the structure of the composite. However, for displacement greater than 0.8 mm, it seems the linearity response no longer holds. This response was observed on each of the six cycles. Further studies are needed to understand the interaction between the fabric and the fiber for large tension displacement. The final sensor pattern was fabricated by using the polyimide fiber textile combination. The pattern design used for the smart textile is shown in Figure 2. The fabric was 0.91 m long and 0.30 m wide. The fiber was routed by using a U-shape format with a 5-cm space between each fiber. There were a total of three curvature points and four straight sections.

The Boeing team in St. Louis fabricated composite panels for testing while incorporating the XP414 textile with the fiber provided ADFORS-Saint-Gobain and the University of Massachusetts, Lowell. The sensing textile was embedded in the composite sample, which was cured by using an autoclave process. The composite material (BMS8-276) is commercially available but has proprietary Boeing specifications.

## 3. DFOST Test Setup

To validate the performance of the embedded sensors, static and dynamic loads were performed by using a composite panel of size 3 ft by 3 ft. In addition to the DFOST sensor embedded in the composite, two additional sensors were added to the surface of the composite. One sensor corresponds to a second distributed fiber sensor placed in the surface (SF), which was glued to the composite by using epoxy. This sensor followed the same pattern as the embedded fiber (EF). The third sensor corresponded to four strain gauges (SG). They were placed at locations 1.57 m, 2.46 m, 3.34 m, and 4.24 m with respect to the surface fiber and locations 1.48 m, 2.48 m, 3.57 m, and 4.58 m with respect to the embedded fiber.

The DFOST embedded in the composite was tested by using a cantilever test approach. The composite sample was placed in between a table and a structural beam and fixed by using clamps. Additionally, a loading string was added at the opposite end of the composite panel to add the load (Figure 2). Static and dynamic tests were performed to determine the efficiency of the smart textile. For the static test, weights were added at a step of 1 kg up to a maximum load of 4 kg. The load was held for approximately 15 s at each loading step before adding the next load. Each test consisted of loading and unloading the composite sample, while measurements were taken by using the OFDR and strain gauge DAQ system. The test was repeated three times to evaluate the repeatable response of the sensors. For the dynamic test, the free composite section was compressed by 1 cm and released. This will allow the sample to move back and forth, generating an oscillation that was captured by the different sensors.

## 4. Results

### 4.1. Static Test

**Figure 3:**
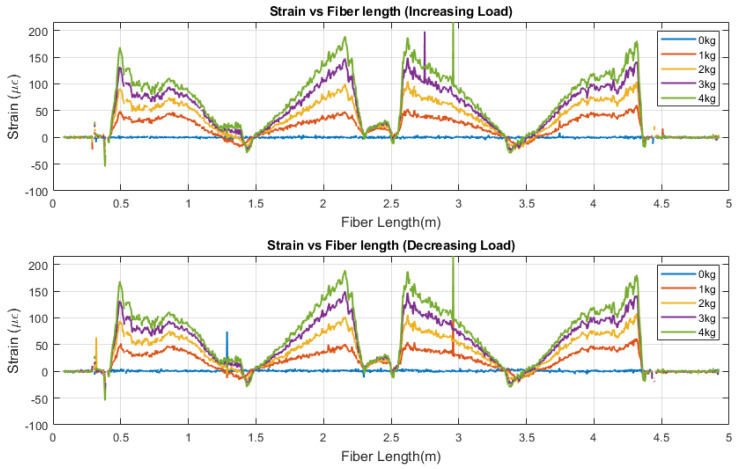
Distributed strain response along the entire fiber length.

As mentioned, strain measurements were collected by using three sensors (embedded fiber, surface fiber, and strain gauges). The data provided by the fiber sensors corresponds to a distributed measurement, meaning that strain data points were collected at different spatial resolutions throughout the entire length of the fiber. For this research, the resolution was set to 2.6 mm. Figure 3 shows the distributed response for the embedded fiber sensor at different loading conditions. A specific pattern is observed related to the fiber optic sensor orientation. For instance, the data located between lengths 0.2 m and 1.5 m shows a decrease in the strain values. This response originates because the fiber is moving away from the fixed point in the cantilever, whereas the maximum strain response happens. The contrary happens when the fiber returns and moves toward the cantilever-fixed point. As a result, the data between 1.5 m and 2.2 m experienced a strain increase. This pattern is repeated twice because there are three loops in the design (Figure 2).

The EF was compared with the SG sensors located at the surface of the composite. Table 1 shows the average and standard deviation for the loading and unloading data at four locations along the fiber. The table shows that the largest standard deviation for the embedded fiber occurs when no loading is applied. This value is expected because the system repeatability at zero strain and spatial resolution of 2.6 mm is <±5 mm according to the data sheet of the equipment. Additionally, the influence of the environmental temperature will affect the strain measurement. Therefore, further investigation must be performed to decouple the temperature and strain response.

**Figure 4:**
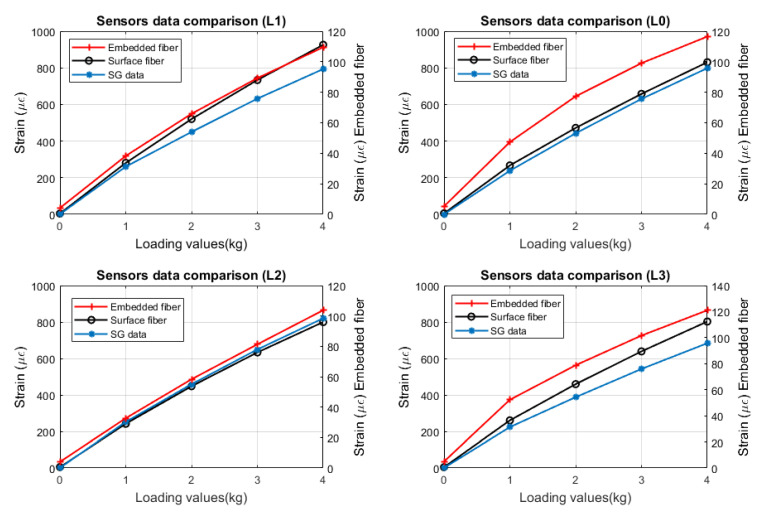
Dataset comparison for different types of sensors.

Comparing the mean values across sensors, the surface fiber and the strain gauge show better agreement (Figure 4). This is because both sensors are positioned on the surface of the composite panel, therefore measuring only surface strain. When looking at the data from the EF, the SF is an average of 6.73 times greater than the EF. The difference between both sensors can be explained by applying bending stress analysis. Knowing the bending moment at any location of the composite panel, the bending stress over the composite’s cross-section can be calculated. The bending moment varies over the height of the cross-sections according to the flexure formula (2), wherein *M* is the bending moment at the location of interest along the composite length, $I_{c}$ is the centroidal moment of inertia of the composite’s cross-section, and *y* is the distance from the composite’s neutral axis to the point of interest along the height of the cross-section. The bending stress is zero at the composite’s neutral axis. The bending stress increases linearly away from the neutral axis until the maximum values at the extreme fibers are at the top and bottom of the composite panel:

(2) $$\sigma_{b}=\frac{-My}{I_{c}}.$$

Once the stress values are known, the strain can be computed by dividing the stress value by the elastic modulus. Because the stress is expected to be at maximum at the top and bottom part of the composite panel and the stress is directly proportional to strain, the strain will also be at maximum in those locations. Because the fiber optic textile was embedded into the composite samples, it was expected for the measured strain to be smaller in comparison to the SF and SG sensor. This hypothesis is demonstrated in Figure 4, wherein the plots show the response at L1, L2, L3, and L4 locations. These locations correspond to the strain gauge location (45 cm away from the cantilever’s nonfixed edge location. As seen in Figure 4 for the same applied load, both the EF and SF read completely different strain values. For example, for a 4-kg load, the EF measures approximately 120 μϵ] whereas the SF reads approximately 900 μϵ]. Despite this difference, the ratio strain between the EF and SF should remain constant across different load values, as discussed previously. Table 1 shows the ratio EF/SF of the mean values for different loading. At a 4-kg load, there is no standard deviation because of how the test was performed. Because the loading was done in an increasing matter, once 4 kg is reached, the same strain information is reflected for the loading and unloading case. Hence, the mean value is the same, yielding no standard deviation. These results demonstrate the ability of the smart textile to measure strain changes inside the composite sample.

The response of the fiber sensors was also compared with SG, as shown in Figure 4. Specifically, the SG measurements were compared with the surface fiber due to the proximity between both sensors. However, the same approach is used to compare the EF, and the SF can be used to compare the response between the SG and the EF. From Figure 4, we observed a good agreement between the SG and SF at locations L0 and L2. For L1 and L3, a difference is seen between both measurements. This difference occurs because of the bonding between the SG and the composite panel. Extra epoxy was required to create a solid bond between the SG and composite panel since locations L1 and L3 did not adhere to the surface correctly. Because of this, a thicker epoxy layer may have been created between the SG and panel, reducing the strain transfer. Hence, a lower strain value should be measured by the SG.

### 4.2. Dynamic Test

**Figure 5:**
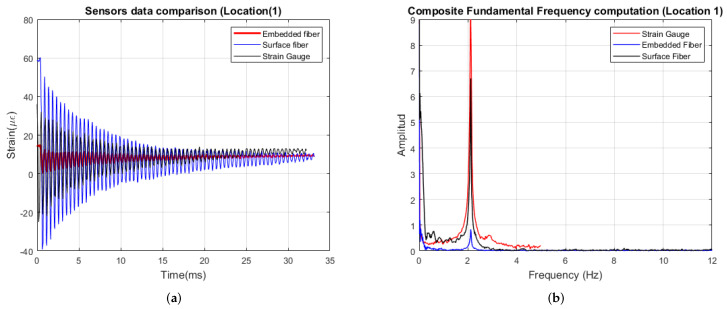
(**a**) Dynamic response response at location 1. (**b**) Frequency response.

The dynamic test was collected by allowing the composite panel to oscillate freely. By using the same cantilever setup, the panel was compressed 1 cm in the Y-axis and released. As the panel oscillated, measurements were collected from the three sensors installed. Figure 5a shows the damping response for location 1. The embedded and surface sensors measured a minimum strain of 3.1 μϵ] and 5.4 μϵ], respectively. The Fourier transform was computed to determine the fundamental frequency estimated to be 2.11 Hz for the induced vibration (Figure 5b).

## 5. Conclusions

This paper demonstrated the integration of distributed fiber optic sensing into a textile by using the embroidering method. By using this approach, the overall installation time of the DFO sensors can be reduced. Additionally, different sensor patterns can be designed to be customized for the customer application. The paper also studied the sensor’s response when the strain was applied. It explored the strain transfer between a polyimide coating and jacketed fiber. As demonstrated during the calibration process, polyimide fiber had a better response when the strain was applied. In addition, the DFOST was embedded into a composite sample. Static and dynamic tests were performed by using a cantilever setup. The sensor measured the strain inside the composite with the largest standard deviation (0.57) when no load was applied and the minimum (0.01) was at 3 kg. The DFOST also measured dynamic vibration with the minimum strain difference recorded value of 3 μϵ] and a fundamental frequency of 2.11 Hz. The measurement values were compared with a fiber sensor located at the surface of the composite panel. We observed a constant strain difference of 8.7 times greater across all loading values from the measurement performed by the surface sensor. As explained previously, this difference is that the highest strain values are always observed at the surface of any material.

## Figures and Tables

**Table 1:** Mean and standard deviation at each strain gauge location.

		Mean					Standard Deviation				
**Sensor Location**	**Fiber Location**	**0 kg**	**1 kg**	**2 kg**	**3 kg**	**4 kg**	**0 kg**	**1 kg**	**2 kg**	**3 kg**	**4 kg**
0	EF	3.28	43.95	71.85	93.09	110.08	0.57	0.44	0.3	0.01	-
	SF	3.6	263.86	465.46	651.94	821.23	0.68	1.16	5.1	5.32	-
	SG	3.308	216.89	399.74	567.61	721.08	0.23	0.51	0.54	0.17	-
	EF/SF	-	0.166	0.154	0.142	0.134	-	-	-	-	-
1	EF	1.97	34.89	62	82.33	101.39	0.46	0.43	0.55	0.66	-
	SF	5.45	275.45	509.68	720.44	910.08	0.41	0.48	1.1	8.22	-
	SG	5.421	262.6	458.36	638.1	801.91	0.35	0.42	0.63	0.49	-
	EF/SF	-	0.12	0.12	0.11	0.11	-	-	-	-	-
2	EF	2.494	29.833	54.25	75.69	96.81	0.36	0.015	0.14	0.24	-
	SF	−2.21	226.81	427.84	616.69	790.22	0.4	1.65	4.42	17.31	-
	SG	5.83	254.05	461.45	650.79	820.28	0.31	0.46	0.3	0.56	-
	EF/SF	-	0.131	0.126	0.122	0.122	-	-	-	-	-
3	EF	1.345	47.37	74.48	94.5	112.64	0.55	0.48	0.25	0.22	-
	SF	3.53	256.21	454.5	633.15	797	0.54	1.19	3.07	7.14	-
	SG	−2.96	214.27	381.5	536.46	683.87	0.376	1.02	0.99	2.42	-
	EF/SF	-	0.184	0.163	0.149	0.141	-	-	-	-	-

## Data Availability

Not applicable.
